# Association of HbA1c/HDL-C ratio and depression with cardiometabolic multimorbidity in middle-aged and older adults: a nationwide prospective cohort study

**DOI:** 10.3389/fnut.2025.1642243

**Published:** 2025-10-02

**Authors:** Jiayang Dong, Aodi Huang, Zhiqiang Zhang, Xinyue Yang, Jiayi Sun, Wenjuan Zhang

**Affiliations:** ^1^Department of Cardiology, Tianjin Medical University General Hospital, Tianjin, China; ^2^Department of Cardiology, Zhengzhou Central Hospital Affiliated to Zhengzhou University, Zhengzhou, China; ^3^Department of Cardiology, Tianjin Chest Hospital, Tianjin, China

**Keywords:** glycosylated hemoglobin A1c, high-density lipoprotein cholesterol, HbA1c/HDL-C ratio, depression, cardiometabolic multimorbidity, CHARLS

## Abstract

**Background:**

Cardiometabolic multimorbidity (CMM)—the coexistence of two or more cardiometabolic diseases such as diabetes, heart disease, and stroke—poses serious health risks. Depression has recently been recognized as an independent risk factor for CMM. However, the role of the glycosylated hemoglobin A1c to high-density lipoprotein cholesterol ratio (HbA1c/HDL-C) in predicting CMM remains unclear. This study investigates the independent and combined effects of the HbA1c/HDL-C ratio and depression on CMM risk in middle-aged and older adults.

**Methods:**

Data were drawn from the China Health and Retirement Longitudinal Study (CHARLS) from 2011 to 2018, including 7,256 participants aged 45 and above. Kaplan–Meier analysis estimated cumulative CMM incidence. Multivariable Cox proportional hazards models assessed the independent and joint effects of the HbA1c/HDL-C ratio and depression on CMM risk. Restricted cubic splines evaluated potential nonlinear relationships. Receiver operating characteristic (ROC) analysis compared the predictive performance of the HbA1c/HDL-C ratio, depression, and their combination. Subgroup and sensitivity analyses tested the robustness of findings.

**Results:**

Over the 7-year follow-up, 419 participants (5.77%) developed CMM. Higher HbA1c/HDL-C ratios and depression were associated with greater cumulative incidence of CMM. The HbA1c/HDL-C ratio was positively associated with CMM risk, showing a nonlinear trend. Participants with both a high HbA1c/HDL-C ratio and depression had significantly increased CMM risk (HR = 2.59, 95% CI: 1.82–3.68) compared to those with either factor alone. Combined analysis consistently outperformed single predictors in ROC analysis. Subgroup and sensitivity analyses confirmed the robustness of results. Depression did not mediate the HbA1c/HDL-C–CMM association.

**Conclusion:**

Both the HbA1c/HDL-C ratio and depression are independently associated with CMM. Depression did not mediate the association between the HbA1c/HDL-C ratio and CMM. Their combined effect substantially increases CMM risk, underscoring the importance of integrating metabolic and psychological assessments in early identification and personalized prevention strategies for high-risk populations.

## Graphical abstract

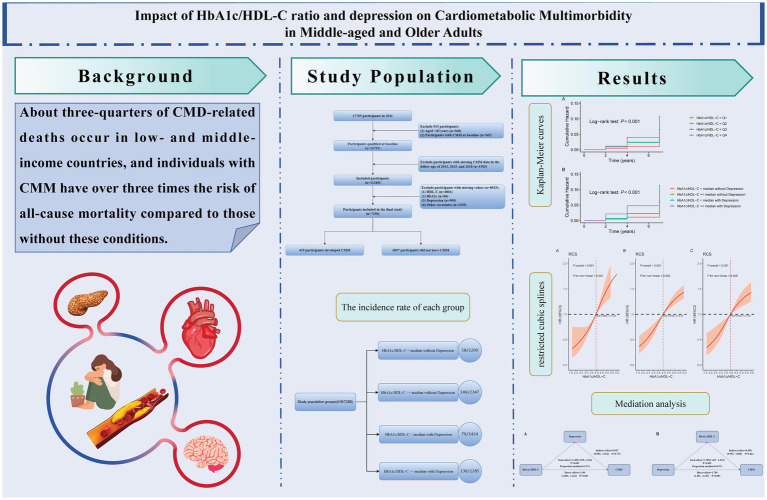

## Background

Cardiometabolic multimorbidity (CMM) refers to the coexistence of two or more cardiometabolic diseases (CMDs), such as diabetes, heart disease, and stroke (1), which are major contributors to global mortality and disability (2). It is estimated that three-quarters of deaths related to CMDs occur in low- and middle-income countries, with 30% classified as premature deaths (3). Previous studies have shown that individuals with CMM have more than three times the risk of all-cause mortality compared to those without CMDs (4), along with a 2- to 5-fold increase in dementia risk (5, 6). In a study involving Chinese adults aged 18 years and older, the prevalence of CMM was found to increase with age, reaching 5.2% in individuals aged 40 years and older and 11.6% in those aged 60 years and older (7). The multimorbidity nature of CMM complicates disease management, as optimal treatment strategies may differ among patients, necessitating a more comprehensive and individualized management approach (8). Given the high prevalence of CMM and its associated disease burden, early identification of high-risk individuals is crucial for optimizing risk stratification and developing personalized treatment plans.

In recent years, overweight and obesity have been recognized as significant risk factors for CMM. Numerous prospective cohort studies from the United States and Europe indicate that individuals who are overweight or obese have a significantly increased risk of developing CMM compared to those with a healthy weight (1). Common obesity measurement indicators, such as body mass index (BMI), waist circumference (WC), and waist-to-height ratio, have been widely used to assess metabolic risk. Recent research has shown that the ratio of non-high-density lipoprotein cholesterol (non-HDL-C) to high-density lipoprotein cholesterol (HDL-C) and the visceral adiposity index outperform traditional lipid parameters—such as total cholesterol (TC), low-density lipoprotein cholesterol (LDL-C), and triglycerides (TG)—in predicting CMM (9, 10). However, these indicators primarily reflect lipid metabolism, while there exists a complex interplay between glucose and lipid metabolism. Studies have demonstrated that lipid metabolism abnormalities are not only a consequence of diabetes but may also contribute to glucose metabolic disorders (11). Therefore, the glycosylated hemoglobin A1c to high-density lipoprotein cholesterol (HbA1c/HDL-C) ratio has been proposed as a comprehensive measure of glucose-lipid metabolism (12). HbA1c reflects average blood glucose levels over the past 2 to 3 months and is widely used in diabetes management (13). Moreover, its levels exhibit a J-shaped relationship with cardiovascular disease (CVD) and all-cause mortality, with HbA1c levels >8.0% significantly increasing the risk of CVD and death (14). On the other hand, HDL-C plays multiple biological roles, including cholesterol reverse transport, anti-inflammatory and anti-thrombotic properties, and promoting insulin secretion (15); it is negatively correlated with the risk of atherosclerotic cardiovascular disease (16). Data from prospective cohort studies in China have indicated that higher HbA1c/HDL-C ratios are significantly associated with an increased risk of carotid atherosclerosis in older adults (12). This finding further suggests that the HbA1c/HDL-C ratio could serve as a comprehensive biomarker for glucose-lipid metabolic disorders, providing new insights for the early identification and risk assessment of CMM.

Additionally, depression has been confirmed as an independent risk factor for CMM, adversely affecting the progression from health to CMM (17). Previous meta-analyses have shown that individuals with depression face a significantly increased risk of myocardial infarction (MI) and coronary artery disease (18). Furthermore, a prospective cohort study found that the risk of developing CMM is 31% higher in individuals with depression compared to those without (19). Data from the China Kadoorie Biobank have shown that individuals who experienced severe depression in the past year had significantly higher risks of stroke, ischemic heart disease, and type 2 diabetes, even after adjusting for major confounding factors (20–22). These findings indicate that depression impacts not only individual mental health but may also accelerate the onset and progression of various chronic diseases through complex physiological and behavioral mechanisms.

Glucose-lipid metabolic disorders and depression have both been independently associated with an increased risk of CMM. Emerging evidence also suggests a potential bidirectional relationship between these two conditions (23), and their coexistence may exert synergistic or additive effects on CMM risk. However, few studies have systematically examined the association between the HbA1c/HDL-C ratio and CMM risk, particularly regarding its joint effect with depressive symptoms. Given the heightened vulnerability of middle-aged and older adults to both metabolic dysfunction and psychological distress, this study uses data from the China Health and Retirement Longitudinal Study (CHARLS) to investigate the independent effect of the HbA1c/HDL-C ratio and its joint effect with depression on CMM risk. The findings are expected to enhance understanding of how metabolic and psychological factors jointly contribute to CMM development and to inform early identification and targeted interventions for high-risk populations.

## Methods

### Data source and study population

The CHARLS is a nationwide, representative longitudinal survey focusing on individuals aged 45 and older. The baseline survey was conducted between 2011 and 2012, with follow-up assessments in 2013, 2015, 2018, and 2020. The CHARLS employs computer-assisted personal interviewing technology to systematically collect data on respondents’ demographics, health status, lifestyle, socioeconomic conditions, and family support. During each follow-up, researchers perform physical measurements and health function tests, including blood pressure, lung capacity, grip strength, and balance ability. Additionally, blood samples are collected for biochemical analysis to assess important health indicators such as blood glucose and cholesterol levels (24). All participants provided written informed consent, and the study received ethical approval from the Biomedical Ethics Committee of Peking University (IRB 00001052-11015). This study strictly adheres to the STROBE guidelines for reporting observational epidemiological research, ensuring scientific rigor in study design and data collection (25).

**Figure 1:**
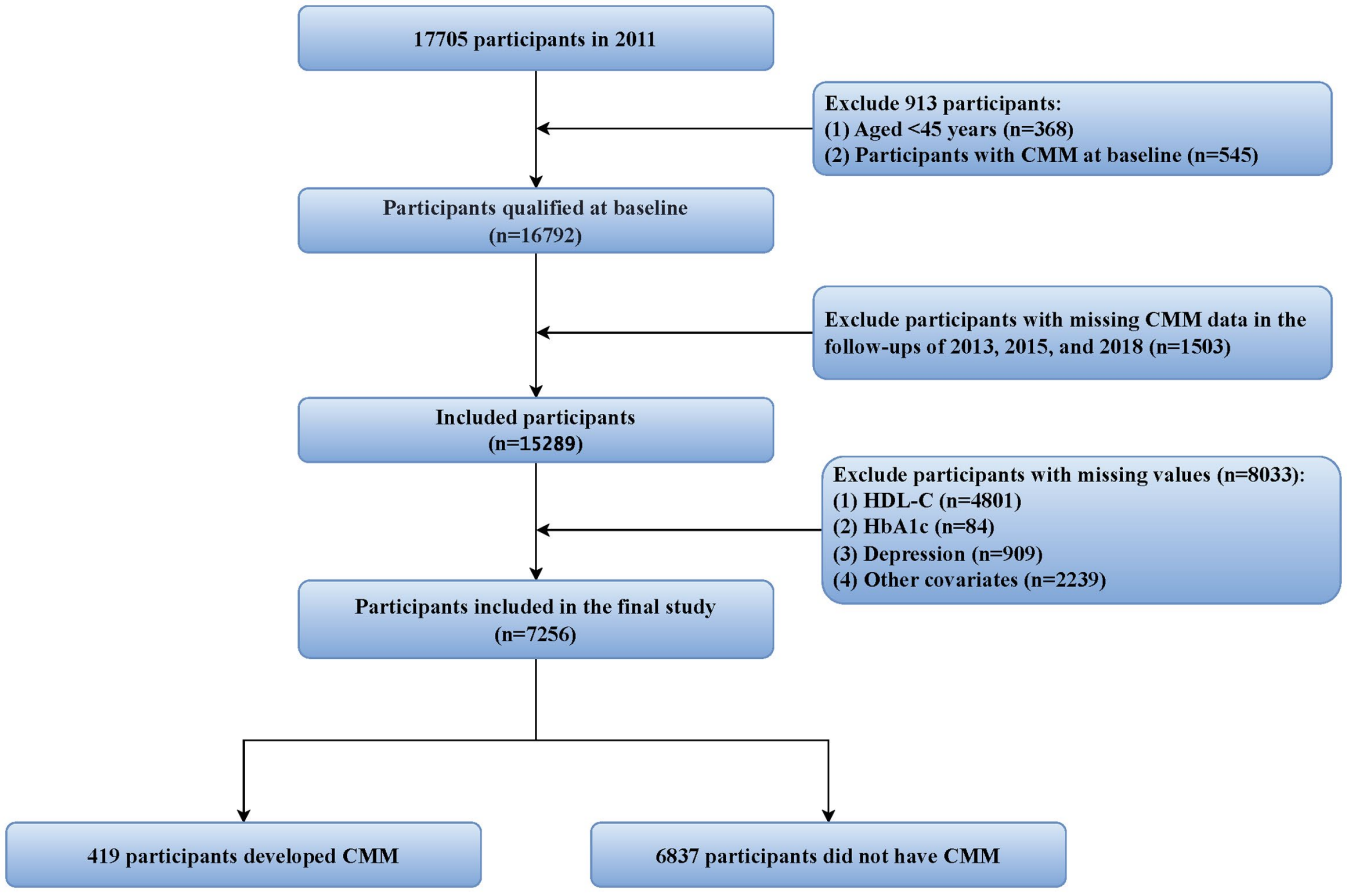
Flowchart of the study population selection process. CMM, cardiometabolic multimorbidity; HDL-C, high-density lipoprotein cholesterol; HbA1c, hemoglobin A1c.

For this research, the 2011 CHARLS data was utilized as baseline data, with follow-ups conducted in 2013, 2015, and 2018 to assess study outcomes. To ensure data integrity and research rigor, the following exclusion criteria were established: (1) participants younger than 45 years; (2) individuals with missing CMM data or already diagnosed with CMM during the 2011 baseline survey; (3) participants with missing data on HbA1c, HDL-C, or any covariates included at baseline; and (4) individuals with missing CMM data during follow-ups in 2013, 2015, or 2018. Ultimately, 7,256 participants who met the study criteria were included, with the specific inclusion and exclusion processes detailed in Figure 1.

### Data collection and measurement

Trained staff from the Chinese Center for Disease Control and Prevention collected three tubes of venous blood from each respondent, requiring overnight fasting; however, if fasting was not achieved, blood samples were still collected, and fasting status was recorded. The collected blood samples were preserved by freezing and transported to the Clinical Laboratory of Capital Medical University for centralized testing. Specific tests included TG, TC, LDL-C, and HDL-C, assessed using enzymatic colorimetric methods. HbA1c was measured using boronate affinity high-performance liquid chromatography; creatinine was assessed using the rate-blanked and compensated Jaffe method; uric acid (UA) was measured with the UA Plus method; and C-reactive protein (CRP) was evaluated using immunoturbidimetric assays. All results were merged and verified by two staff members from the Chinese Center for Disease Control and Prevention to ensure accuracy and consistency (26). Height was measured with a stadiometer, with participants standing upright, heels together, and weight evenly distributed. Weight was assessed using a calibrated scale with participants barefoot. BMI = weight (kg)/height (m)^2^ (9). The HbA1c/HDL-C ratio was calculated using the following formula (12):


$$\mathrm{HbA}1c/\mathrm{HDL}-C\;\text{ratio}=\frac{\mathrm{HbA}1c\;(\%)}{\mathrm{HDL}-C\;(\text{mmol}/L)}$$

Depressive symptoms were assessed using the Chinese version of the 10-item Center for Epidemiological Studies Depression Scale (CES-D) (Supplementary Table S1). Each item was scored on a 4-point scale ranging from 0 to 3, with the total score ranging from 0 to 30. A total score of 10 or above was used to indicate the presence of depression, with higher scores reflecting greater severity of depressive mood (27).

### Assessment of CMM events

The primary outcome of this study was the occurrence of CMM, defined as the coexistence of two or more CMDs, including stroke, heart disease, and diabetes (28). Heart disease and stroke diagnoses were based on standardized survey questions: “Have you ever been diagnosed by a doctor with heart attack, coronary heart disease, angina, congestive heart failure, or other heart problems?” and “Have you ever been diagnosed by a doctor with stroke?” Diabetes was diagnosed if any of the following criteria were met: fasting blood glucose level ≥126 mg/dL (7 mmol/L), random blood glucose level ≥200 mg/dL (11.1 mmol/L), HbA1c level ≥6.5%, currently using diabetes medication, or self-reported diagnosis of “Have you ever been diagnosed by a doctor with diabetes or high blood sugar?” based on the standards published by the American Diabetes Association in 2024 (29). Although the current diagnostic criteria were published in 2024, the core thresholds have remained unchanged since 2010 (30). Thus, our classification of baseline data (2011–2012) is consistent with both historical and current standards. The event time was defined as the interval between the baseline assessment date and the date of the first reported occurrence of CMM. Once diagnosed with CMM, participants were no longer followed up. For individuals who did not report CMM during the follow-up period, the duration of follow-up was determined from the baseline assessment date to the final follow-up date.

### Covariates

We selected covariates based on previously published literature and the availability of variables in the CHARLS database (9, 10, 31, 32). Basic information was collected from participants via structured questionnaires, including demographic factors such as gender, age, location (categorized as urban and rural), marital status (categorized as single and married, with married defined as living with a spouse), and education level (categorized as illiteracy, primary school, middle school, and high school and above). Additionally, various health-related indicators were collected, including WC, BMI, smoking (categorized as non-smoker, ex-smoker, and smoker), drinking (defined as drinking more than once a month), systolic blood pressure (SBP), diastolic blood pressure (DBP), sleep duration (indicating actual nighttime sleep time), hypertension, kidney disease, antidiabetic, antidyslipidemic, LDL-C, TC, TG, creatinine, UA, and CRP. Hypertension was defined as SBP ≥ 140 mmHg or DBP ≥ 90 mmHg, self-reported diagnosis of hypertension, or currently using antihypertensive medication (33). The diagnosis of kidney disease primarily relied on self-reported history of prior kidney disease.

### Statistical analysis

We first assessed the proportion of missing data for each selected variable and excluded those with more than 20% missing values. To evaluate multicollinearity, we then calculated the generalized variance inflation factor (GVIF) for the remaining variables. Variables with GVIF values exceeding 10 were removed, and the rest were retained for inclusion in the final analysis (34). This study first described baseline data according to CMM status, HbA1c/HDL-C ratio quartiles, and depression status. The normality of continuous variables was assessed using the Anderson-Darling test and Q-Q plots. Due to the non-normal distribution of the data, continuous variables were expressed as medians and interquartile ranges (IQR), with group comparisons conducted using the Mann–Whitney U test. Categorical variables were presented as counts and percentages, with comparisons between groups using the chi-squared test or Fisher’s exact test. Given the lack of clear clinical cutoff values for the HbA1c/HDL-C ratio, the median was utilized as the threshold based on prior research (12). To explore the joint effect of the HbA1c/HDL-C ratio and depression on the risk of CMM, participants were divided into four groups: (1) HbA1c/HDL-C < median without depression, (2) HbA1c/HDL-C ≥ median without depression, (3) HbA1c/HDL-C < median with depression, and (4) HbA1c/HDL-C ≥ median with depression. Kaplan–Meier survival analysis was employed to estimate the cumulative incidence of CMM for each group, with cumulative risk curves plotted and intergroup differences compared using the log-rank test.

Multivariable-adjusted Cox proportional hazards models were employed to examine the associations of baseline HbA1c/HDL-C ratio with CMM, both independently and jointly with depression. The HbA1c/HDL-C ratio was analyzed as quartiles and as a continuous variable. For all analyses, three models were specified: Model 1 without adjustment, Model 2 adjusted for demographic and lifestyle factors (gender, age, location, marital status, education, BMI, WC, smoking, drinking, SBP, DBP, and sleep duration), and Model 3 further adjusted for clinical variables (hypertension, kidney disease, antidiabetic, antidyslipidemic, LDL-C, TC, TG, creatinine, UA, and CRP).

Furthermore, to explore the potential nonlinear relationship between the HbA1c/HDL-C ratio and the risk of CMM, restricted cubic splines (RCS) were constructed based on Cox regression models. To compare the predictive abilities of the HbA1c/HDL-C ratio, depression, and their combination on CMM risk, receiver operating characteristic (ROC) curves were constructed at two, four, and seven-year time points to assess predictive performance.

To explore the effect of the individual HbA1c/HDL-C ratio and its joint effect with depression on the risk of CMM in different subgroups, we performed subgroup and interaction analyses. The calculation of interactions was performed using product terms in the main analysis. We further conducted sensitivity analyses to enhance the reliability and robustness of our findings. First, participants with a history of heart disease, stroke, or diabetes at baseline were excluded to reduce potential confounding from pre-existing conditions. Second, to address potential reverse causality, individuals who developed CMM during the 2013 and 2015 follow-up waves were excluded, ensuring a more accurate temporal relationship between exposure and outcome. Finally, to handle missing data in a methodologically sound manner, we employed multiple imputation using chained equations (MICE). This approach allowed for the preservation of statistical power and minimized bias associated with missing values.

We conducted counterfactual-based mediation analyses using the cmest function to examine the direct and indirect relationships among the HbA1c/HDL-C ratio, depression, and the risk of developing CMM. First, we assessed whether depression mediated the association between the HbA1c/HDL-C ratio and CMM using a Cox model for the outcome and a logistic model for the binary mediator, adjusting for all covariates. Second, we reversed the model to evaluate whether the HbA1c/HDL-C ratio mediated the relationship between depression and CMM, applying a linear model for the continuous mediator and a Cox model for the outcome, with the same covariate adjustments. Both analyses used imputation-based estimation with 1,000 bootstrap resamples to derive the total effect, total natural direct effect, and total natural indirect effect with 95% confidence intervals (CIs). A *p*-value < 0.05 for the TNIE was considered evidence of significant mediation. Hazard ratios (HRs) and 95% CIs were reported for all effects. All statistical analyses were performed using R software (version 4.3.2), with a two-sided significance level set at *p* < 0.05.

## Results

### Baseline characteristics

Taking into account the proportion of missing values and the results of the GVIF assessment, a total of 22 covariates were ultimately included in the final analysis (Supplementary Tables S2, S3). A total of 7,256 participants from the 2011 to 2018 waves of the CHARLS were included in this study. The mean age was 58 years, 52.83% were female, and 65.99% resided in rural areas. During the 7-year follow-up period, 419 incident cases of CMM were identified, corresponding to an incidence proportion of 5.77%. Compared with participants without CMM, those with CMM were older, more often female, had higher BMI and WC, were more likely to have depression and hypertension, and exhibited higher use of antidiabetic and antidyslipidemic medications as well as elevated lipid and inflammatory markers (Table 1). Stratification by HbA1c/HDL-C quartiles showed that higher ratios were associated with male sex, urban residence, higher BMI, smoking, hypertension, and increased use of metabolic medications (Supplementary Table S4). Participants with depression were more likely to be female, older, rural residents, single, and have lower educational attainment and HbA1c/HDL-C ratios (Supplementary Table S5).

**Table 1:** Baseline characteristics of the included population.

Characteristics	Overall (*n* = 7,256)	Non-CMM (*n* = 6,837)	CMM (*n* = 419)	*p* value
Gender, *n* (%)				0.005
Female	3,833 (52.83)	3,583 (52.41)	250 (59.67)	
Male	3,423 (47.17)	3,254 (47.59)	169 (40.33)	
Age (years)	58.00 [52.00, 65.00]	58.00 [51.00, 65.00]	60.00 [55.00, 66.00]	<0.001
Location, *n* (%)				0.458
Urban	2,468 (34.01)	2,318 (33.90)	150 (35.80)	
Rural	4,788 (65.99)	4,519 (66.10)	269 (64.20)	
Marital, *n* (%)				0.682
Single	1,118 (15.41)	1,050 (15.36)	68 (16.23)	
Married	6,138 (84.59)	5,787 (84.64)	351 (83.77)	
Education, *n* (%)				0.132
Illiteracy	1986 (27.37)	1865 (27.28)	121 (28.88)	
Primary school	3,045 (41.97)	2,867 (41.93)	178 (42.48)	
Middle school	1,486 (20.48)	1,417 (20.73)	69 (16.47)	
High school and above	739 (10.18)	688 (10.06)	51 (12.17)	
BMI (kg/m^2^)				<0.001
<24	4,366 (60.17)	4,199 (61.42)	167 (39.86)	
≥24	2,890 (39.83)	2,638 (38.58)	252 (60.14)	
WC (cm)	84.00 [77.30, 91.20]	84.00 [77.00, 91.00]	90.00 [82.20, 98.00]	<0.001
Smoking, *n* (%)				0.001
Non-smoker	4,390 (60.50)	4,114 (60.17)	276 (65.87)	
Ex-smoker	628 (8.65)	582 (8.51)	46 (10.98)	
Smoker	2,238 (30.84)	2,141 (31.31)	97 (23.15)	
Drinking, *n* (%)	1821 (25.10)	1736 (25.39)	85 (20.29)	0.023
SBP (mmHg)	126.33 [114.00, 141.33]	126.00 [113.67, 140.67]	134.33 [122.00, 150.67]	<0.001
DBP (mmHg)	74.33 [67.00, 83.00]	74.33 [67.00, 82.67]	78.00 [70.33, 87.00]	<0.001
Depression, *n* (%)	2,709 (37.33)	2,494 (36.48)	215 (51.31)	<0.001
Sleep duration (h)	6.50 [5.00, 8.00]	7.00 [5.00, 8.00]	6.00 [5.00, 8.00]	0.041
Hypertension, *n* (%)	2,820 (38.86)	2,552 (37.33)	268 (63.96)	<0.001
Kidney disease, *n* (%)	468 (6.45)	424 (6.20)	44 (10.50)	0.001
Antidiabetic, *n* (%)	220 (3.03)	168 (2.46)	52 (12.41)	<0.001
Antidyslipidemic, *n* (%)	347 (4.78)	291 (4.26)	56 (13.37)	<0.001
LDL-C (mmol/L)	2.95 [2.42, 3.54]	2.94 [2.42, 3.52]	3.09 [2.48, 3.76]	0.001
TC (mmol/L)	4.91 [4.32, 5.54]	4.89 [4.31, 5.53]	5.09 [4.46, 5.79]	<0.001
TG (mmol/L)	1.16 [0.83, 1.68]	1.14 [0.82, 1.65]	1.38 [0.99, 2.05]	<0.001
Creatinine (μmol/L)	67.00 [58.00, 78.00]	67.00 [58.00, 78.00]	67.00 [59.00, 78.00]	0.576
UA (μmol/L)	254.40 [211.88, 304.70]	253.80 [211.70, 304.30]	264.30 [216.45, 310.65]	0.060
CRP (mg/L)	0.99 [0.54, 2.07]	0.96 [0.53, 2.00]	1.50 [0.77, 3.00]	<0.001
HbA1c/HDL-C	3.94 [3.23, 4.88]	3.91 [3.21, 4.83]	4.51 [3.71, 5.78]	<0.001

BMI, body mass index; WC, waist circumference; SBP, systolic blood pressure; DBP, diastolic blood pressure; LDL-C, low-density lipoprotein cholesterol; TC, total cholesterol; TG, triglyceride; UA, uric acid; CRP, C-reactive protein; HDL-C, high-density lipoprotein cholesterol; HbA1c, hemoglobin A1c.

### Association between HbA1c/HDL-C ratio and CMM risk during the 2011–2018 follow-up

**Figure 2:**
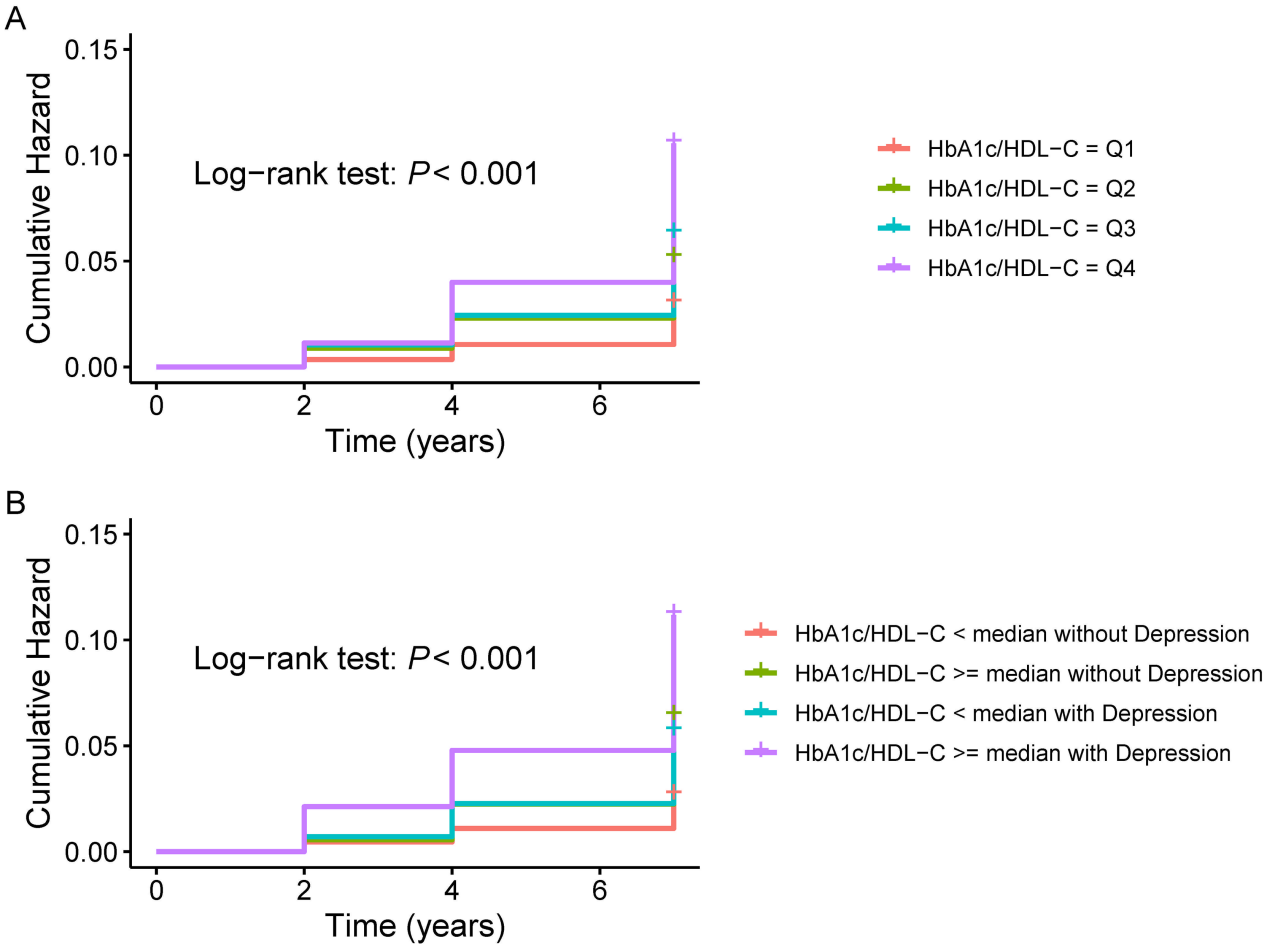
K-M plot of CMM by depression and HbA1c/HDL-C ratio levels. **(A)** K–M plot of CMM by HbA1c/HDL-C ratio levels. **(B)** K-M plot of CMM by the joint effect of HbA1c/HDL-C ratio and depression. CMM, cardiometabolic multimorbidity; HDL-C, high-density lipoprotein cholesterol; HbA1c, hemoglobin A1c.

Figure 2A displays Kaplan–Meier curves showing that CMM incidence increased with higher HbA1c/HDL-C ratio quartiles. To further evaluate the association between the HbA1c/HDL-C ratio and the risk of developing CMM, Cox regression analyses were conducted. In the unadjusted model, individuals in the highest quartile of the HbA1c/HDL-C ratio had a significantly elevated risk of CMM compared to those in the lowest quartile (HR = 3.51, 95% CI = 2.60–4.74). This association remained statistically significant after adjustment for all potential confounders (HR = 2.19, 95% CI = 1.14–3.32). The Schoenfeld residuals test indicated no violation of the proportional hazards assumption (*p* > 0.05) across all models, regardless of whether covariates were adjusted. Furthermore, when the HbA1c/HDL-C ratio was modeled as a continuous variable, each IQR increase in the ratio was associated with a 19% higher risk of CMM after full adjustment (HR = 1.19, 95% CI = 1.12–1.27) (Supplementary Table S6).

### Joint association of HbA1c/HDL-C ratio and depression with the risk of CMM

Figure 2B shows the Kaplan–Meier curves for cumulative CMM incidence by HbA1c/HDL-C ratio and depression. The highest risk was observed in participants with both a high ratio (above the median) and depression. Cox regression models were then used to evaluate the joint effects of HbA1c/HDL-C and depression on CMM risk. In the fully adjusted model, individuals with both a higher HbA1c/HDL-C ratio and depression had the highest risk (HR = 2.59, 95% CI = 1.82–3.68), followed by those with depression but a lower HbA1c/HDL-C ratio (HR = 2.05, 95% CI = 1.45–2.90), and then those with a higher ratio but without depression (HR = 1.57, 95% CI = 1.11–2.21) (Table 2). Across all models, the Schoenfeld residuals test indicated no violation of the proportional hazards assumption (*p* > 0.05).

**Table 2:** The joint effect between HbA1c/HDL-C ratio and depression on the risk of CMM incidence.

Categories	Model 1		Model 2		Model 3	
	HR (95% CI)	*P* value	HR (95% CI)	*P* value	HR (95% CI)	*P* value
HbA1c/HDL-C < median without depression	1.00 (ref)		1.00 (ref)		1.00 (ref)	
HbA1c/HDL-C > = median without depression	2.40 (1.77, 3.25)	<0.001	1.88 (1.37, 2.56)	<0.001	1.57 (1.11, 2.21)	0.010
HbA1c/HDL-C < median with depression	2.14 (1.52, 3.00)	<0.001	2.19 (1.55, 3.09)	<0.001	2.05 (1.45, 2.90)	<0.001
HbA1c/HDL-C > = median with depression	4.20 (3.09, 5.71)	<0.001	3.40 (2.47, 4.67)	<0.001	2.59 (1.82, 3.68)	<0.001

HbA1c, hemoglobin A1c; HDL-C, high-density lipoprotein cholesterol; CMM, cardiometabolic multimorbidity; HR, hazard ratio; ref, reference. Model 1: Crude model; Model 2: Adjusted for age, gender, education, location, marital, BMI, WC, smoking, drinking, SBP, DBP, and sleep duration; Model 3: Further adjusted for hypertension, kidney disease, antidiabetic, antidyslipidemic, LDL-C, TC, TG, creatinine, UA, and CRP.

### Dose–response relationship between HbA1c/HDL-C ratio and CMM

**Figure 3:**
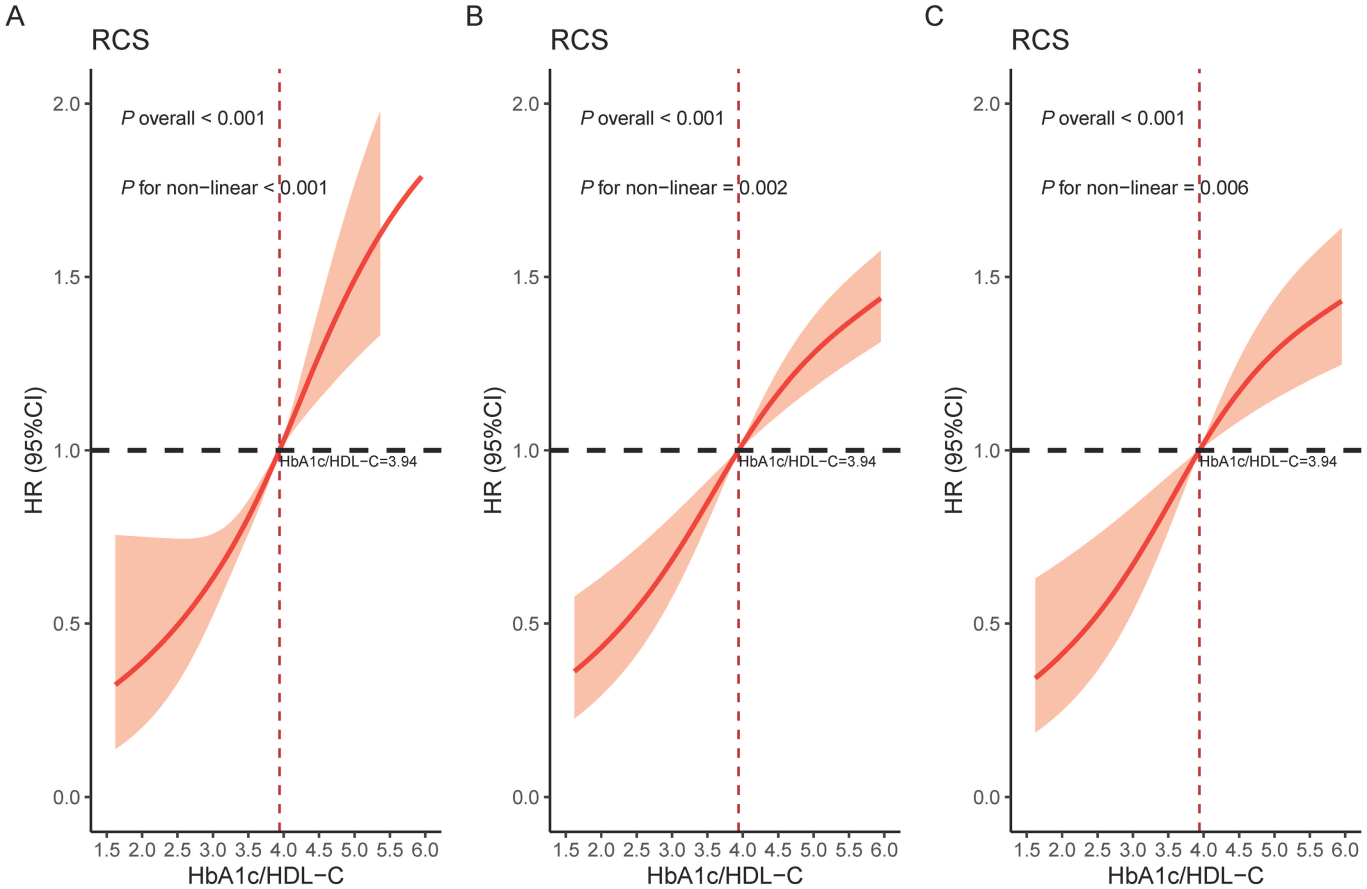
Restricted cubic spline of the association between the HbA1c/HDL-C ratio and the risk of CMM. **(A)** Crude model; **(B)** Adjusted for age, gender, education, location, marital, BMI, WC, smoking, drinking, SBP, DBP, and sleep duration; **(C)** Further adjusted for hypertension, kidney disease, antidiabetic, antidyslipidemic, LDL-C, TC, TG, creatinine, UA, and CRP. HbA1c, hemoglobin A1c; HDL-C, high-density lipoprotein cholesterol; CMM, cardiometabolic multimorbidity; CI, confidence interval; HR, hazard ratio; BMI, body mass index; WC, waist circumference; SBP, systolic blood pressure; DBP, diastolic blood pressure; LDL-C, low-density lipoprotein cholesterol; TC, total cholesterol; TG, triglyceride; UA, uric acid; CRP, C-reactive protein.

Figure 3A displays the results of the unadjusted RCS model, in which four knots were selected based on the lowest Akaike Information Criterion (AIC) value. The specific knot locations—2.45, 3.51, 4.44, and 6.74—were determined automatically using the datadist function. Figures 3B,C show the RCS results after adjusting for covariates, where three knots were selected based on the same AIC criterion. The corresponding knot locations were 3.23, 3.94, and 4.88. The nonlinear relationship between the HbA1c/HDL-C ratio and CMM risk remained significant after adjustment. Based on the Cox proportional hazards model, the HbA1c/HDL-C ratio corresponding to an HR of 1 was identified as 3.94. Notably, we observed that the risk of developing CMM increased significantly when the HbA1c/HDL-C ratio exceeded 3.94, regardless of whether the model was adjusted for covariates (Figure 3).

### Comparison of predictive efficacy of HbA1c/HDL-C ratio, depression, and their joint effect for CMM risk at various time points

**Figure 4:**
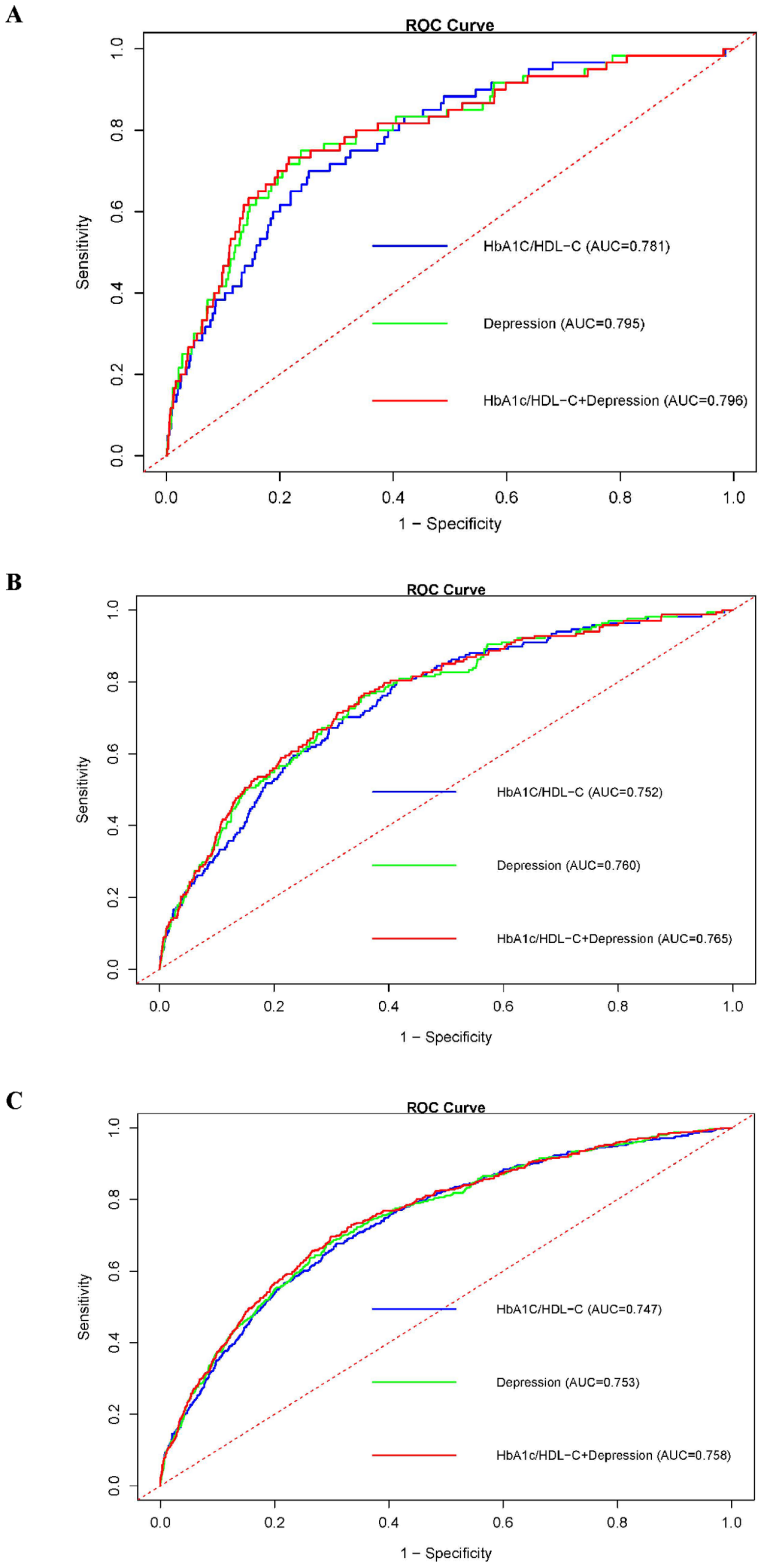
ROC curves comparing the predictive efficacy of the HbA1c/HDL-C ratio, depression, and their joint effect on CMM at different follow-up years. **(A)** ROC curves for prediction in 2013; **(B)** ROC curves for prediction in 2015; **(C)** ROC curves for prediction in 2018. Abbreviations: ROC, receiver operating characteristic; HDL-C, high-density lipoprotein cholesterol; HbA1c, hemoglobin A1c; CMM, cardiometabolic multimorbidity.

After adjusting for all covariates, we evaluated the predictive performance of HbA1c/HDL-C, depression, and their combination for CMM using time-dependent ROC curves at 2-, 4-, and 7-year follow-ups (Figure 4). At 2 years, the AUCs were 0.781 for HbA1c/HDL-C, 0.795 for depression, and 0.796 for the combined model. At 4 years, the AUCs were 0.752, 0.760, and 0.765, respectively; at 7 years, they were 0.747, 0.753, and 0.758. These results indicate that the joint model consistently outperformed either predictor alone. DeLong’s test showed no significant difference between HbA1c/HDL-C and depression alone (*p* = 0.215), but the combined model significantly outperformed HbA1c/HDL-C alone (*p* = 0.012) and depression alone (*p* = 0.001). The joint model also exhibited superior sensitivity and specificity at optimal cut-offs and higher overall prognostic discrimination as measured by the concordance index (Supplementary Table S7).

### Subgroup and sensitivity analyses

**Figure 5:**
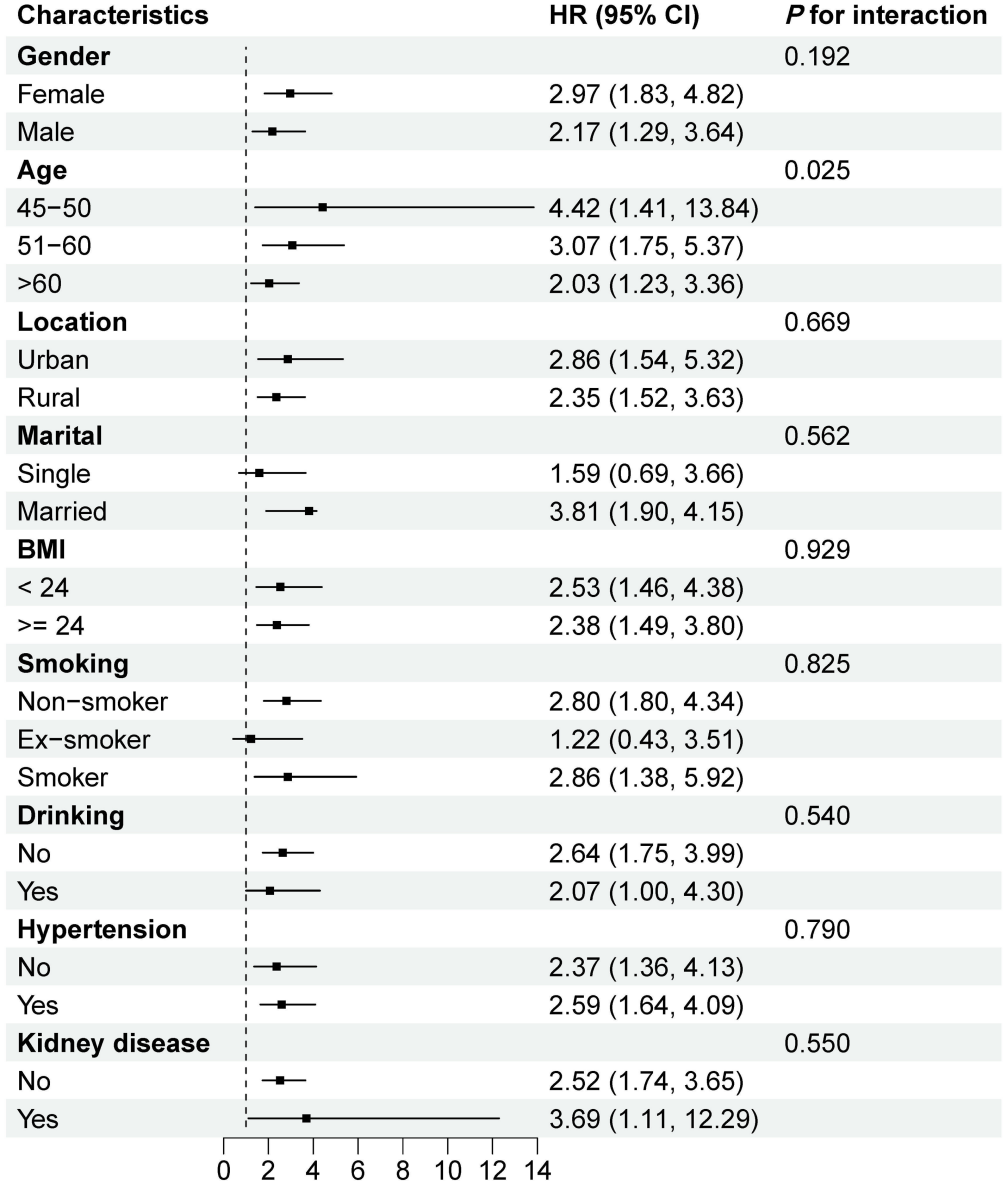
Subgroup and interaction analyses of the combined effect of higher HbA1c/HDL-C ratio and depression on CMM risk. All models were adjusted for age, gender, education, location, marital, BMI, WC, smoking, drinking, SBP, DBP, sleep duration, hypertension, kidney disease, antidiabetic, antidyslipidemic, LDL-C, TC, TG, creatinine, UA, and CRP. HR, hazard ratio; CI, confidence interval; ref., reference; HbA1c, hemoglobin A1c; HDL-C, high-density lipoprotein cholesterol; CMM, cardiometabolic multimorbidity; BMI, body mass index; WC, waist circumference; SBP, systolic blood pressure; DBP, diastolic blood pressure; LDL-C, low-density lipoprotein cholesterol; TC, total cholesterol; TG, triglyceride; UA, uric acid; CRP, C-reactive protein.

Supplementary Figure S1 shows the forest plot of subgroup and interaction analyses for the association between HbA1c/HDL-C ratio and CMM risk. A significant interaction was observed only for age (P for interaction <0.05), with the association remaining positive across all age subgroups. In most other subgroups, the association was also generally positive. Figure 5 illustrates similar analyses for the joint effect of higher HbA1c/HDL-C ratio and depression. Again, age was the only significant effect modifier, while the combined effect remained significant across most subgroups, except among single participants and former smokers. Sensitivity analyses further supported the robustness of our findings. First, excluding participants with pre-existing heart disease, stroke, or diabetes at baseline yielded results consistent with the primary analysis (Supplementary Table S8). Second, excluding individuals who developed CMM during the 2013 or 2015 follow-ups also produced similar results (Supplementary Table S9). Finally, using MICE to handle missing data, rather than simple deletion, did not materially alter the findings (Supplementary Table S10).

### Mediation analysis

Supplementary Figure S2 illustrates the results of the mediation analyses exploring the potential mediating roles of depression and the HbA1c/HDL-C ratio in relation to CMM. In Supplementary Figure S2A, when depression was evaluated as a mediator in the association between the HbA1c/HDL-C ratio and CMM, the indirect effect was not statistically significant (HR = 0.997, 95% CI: 0.984–1.012, *p* = 0.776), despite significant total and direct effects. This suggests that depression does not mediate the relationship between the HbA1c/HDL-C ratio and CMM. Similarly, in Supplementary Figure S2B, when the HbA1c/HDL-C ratio was tested as a mediator between depression and CMM, the indirect effect also failed to reach statistical significance (HR = 0.998, 95% CI: 0.992–1.004, *p* = 0.422). Taken together, these findings indicate that while both depression and the HbA1c/HDL-C ratio are independently associated with increased CMM risk, neither acts as a significant mediator in the pathway of the other.

## Discussion

This study, based on data from the CHARLS, followed 7,256 middle-aged and older Chinese adults over a seven-year period. We found a significant nonlinear association between the HbA1c/HDL-C ratio and the risk of CMM, both as a continuous and categorical variable, even after adjusting for known CMM-related risk factors. Furthermore, the combined presence of a high HbA1c/HDL-C ratio and depression markedly elevated the risk of developing CMM, with the joint effect being stronger than either factor alone. Subgroup and sensitivity analyses further validated the robustness of these findings. In addition, mediation analysis indicated that depression did not mediate the relationship between the HbA1c/HDL-C ratio and CMM risk. Collectively, these findings highlight the potential utility of the HbA1c/HDL-C ratio and depressive symptoms as independent and joint predictors of CMM in this population.

Previous studies have demonstrated that the TG/HDL-C ratio has been recognized as a simple, low-cost, and non-invasive tool for predicting cardiovascular risk, particularly in populations with prediabetes or insulin resistance (35). In parallel, the triglyceride-glucose index is widely used to evaluate insulin resistance and atherosclerosis risk. It has the advantage of being simple to calculate, making it suitable for epidemiological research and community-based screening (25). However, these indicators mainly reflect single metabolic pathways or short-term metabolic states and therefore fail to capture the complex interplay between glucose and lipid metabolism. In contrast, the HbA1c/HDL-C ratio integrates long-term average glycemic levels (HbA1c) with the multiple protective effects of HDL-C, better reflecting the cumulative burden of glucolipid metabolic disorders. Evidence has shown that reducing HbA1c significantly lowers cardiovascular risk among patients with diabetes. For instance, a prospective study involving 4,585 patients with diabetes from England, Scotland, and Northern Ireland reported that each 1% decrease in HbA1c was associated with a 21% reduction in diabetes-related endpoints, a 14% reduction in MI, and a 37% reduction in microvascular complications (36). Likewise, elevated HDL-C levels have consistently been linked to a lower risk of cardiovascular events, even among individuals with LDL-C levels below 70 mg/dL (37, 38). Mechanistically, HDL-C may improve glucose metabolism by enhancing *β*-cell function, insulin secretion, and sensitivity (39). Conversely, hyperglycemia can impair lipolysis, increase free fatty acid synthesis, promote cholesterol accumulation, and disrupt lipid homeostasis, thereby creating a vicious cycle (40). These findings highlight the advantages of the HbA1c/HDL-C ratio as a comprehensive metabolic indicator. Recent studies have further supported its prognostic value in cardiovascular risk assessment. For example, a nationwide prospective cohort study found that a higher HbA1c/HDL-C ratio was significantly associated with increased stroke risk (32). In another study of 384 ST-segment elevation myocardial infarction patients undergoing percutaneous coronary intervention, the HbA1c/ApoA1 ratio (ApoA1 being the major protein component of HDL) was identified as an independent predictor of in-hospital adverse cardiovascular events (41). Consistent with these findings, our study demonstrated that a higher HbA1c/HDL-C ratio was significantly associated with an increased risk of CMM.

A prospective cohort study of 6,663 participants also found that more frequent depression significantly increased the risk of incident CMM, with a 73% increase in risk for every 9-point rise in CES-D-10 score (42). A large-scale meta-analysis also showed that individuals with severe mental disorders had a 53% higher prevalence of CVD, a 78% higher incidence, and an 85% higher risk of CVD-related mortality compared to the general population (43). In our study, Individuals with both a high HbA1c/HDL-C ratio and depressive symptoms had a higher CMM risk than those with either factor alone. The non-significant interaction term (*p* > 0.05) suggests their effects are more likely additive rather than multiplicative. Furthermore, mediation analysis indicated that depression did not mediate the association between the HbA1c/HDL-C ratio and CMM, implying that these factors may influence CMM through independent pathways.

Elevated HbA1c levels reflect not only long-term insulin resistance but also indicate systemic hyperglycemia, lipid metabolism disorders, hypercoagulability, and chronic inflammation (44, 45). HbA1c is widely recognized as a key indicator for the severity of diabetes and CVD. HDL exerts multiple protective effects against atherosclerosis, including inhibition of LDL oxidation (46), stimulation of nitric oxide production via binding to the SR-BI receptor on endothelial cells (47), and reduction of inflammatory cytokines and monocyte adhesion (48). However, these protective mechanisms are often impaired in patients with diabetes, who frequently present with chronic low-grade inflammation and elevated levels of CRP and serum amyloid A, leading to structural and functional alterations in HDL and diminished antioxidant and anti-inflammatory capacities (49, 50). Additionally, increased levels of oxidized lipids within HDL further weaken its ability to protect LDL from oxidative damage (51). These findings reinforce the complex interaction between glucose and lipid metabolism and explain our observation of a nonlinear association between the HbA1c/HDL-C ratio and CMM risk. Notably, the median HbA1c/HDL-C ratio of 3.94, used to dichotomize participants in the joint effect analyses, was also identified by the RCS as a critical inflection point. Beyond this threshold, CMM risk increased significantly, independent of covariate adjustment. This concordance strengthens the robustness of our findings and highlights the potential clinical relevance of 3.94 as a practical threshold for identifying individuals at elevated CMM risk. Future studies should validate this cutoff in diverse populations and assess its feasibility for clinical screening.

The link between depression and CVD risk is multifactorial, involving autonomic dysfunction, inflammation, endothelial dysfunction, and behavioral factors (52). Notably, depression may exhibit acute metabolic suppression (e.g., reduced appetite lowering HbA1c/HDL-C at baseline) but chronically accelerates CMM risk via the hypothalamic–pituitary–adrenal (HPA) axis activation, insulin resistance, and impaired sleep-mediated glucose tolerance (53–55). This temporal, bidirectional effect explains why depressed individuals had lower baseline HbA1c/HDL-C yet higher incident CMM risk.

Recent insights highlight therapeutic strategies targeting these pathways. For example, sodium-glucose co-transporter-2 (SGLT2) inhibitors activate 5′-adenosine monophosphate-activated protein kinase (AMPK) (56). This activation enhances lipid metabolism, suppresses inflammation, and modulates neuroinflammation and HPA axis activity, thereby counteracting both metabolic and psychological drivers of CMM (56). Similarly, natural compounds like curcumin promote autophagy and mitochondrial function, reduce lipid accumulation, improve HDL function, and exert anti-inflammatory and neuroinflammation-modulating effects (57). These multi-mechanistic interventions directly target the dysregulated pathways suggested by our findings linking elevated HbA1c/HDL-C ratios and depressive symptoms to increased CMM risk. Increasing evidence further indicates that multimorbidity represents interconnected, cross-system pathological processes rather than a mere aggregation of single-organ diseases. For instance, liver fibrosis and osteoporosis are closely associated, reflecting shared inflammatory and metabolic pathways that concurrently affect multiple organs (58). Taken together, these observations underscore the systemic nature of CMM and highlight the necessity of considering cross-organ interactions when developing multi-targeted prevention and intervention strategies.

Compared to either the HbA1c/HDL-C ratio or depression, their combined presence demonstrated superior predictive power for CMM, underscoring the value of an integrated approach to risk stratification. Importantly, both HbA1c and HDL-C are readily available from routine blood tests, and depression can be assessed using standard depression scales, making this combined model both practical and clinically applicable.

Subgroup analyses further demonstrated that both the HbA1c/HDL-C ratio alone and its joint effect with depression were consistently associated with increased CMM risk across different stratification factors, highlighting the robustness and broad applicability of the results. However, an exception was observed in the subgroup of former smokers, where no significant association was found between the HbA1c/HDL-C ratio—either alone or in combination with depression—and the risk of CMM. This unexpected result may be explained by two key factors. First, former smokers often adopt healthier lifestyles after cessation, which may mitigate the adverse effects of metabolic dysfunction and psychological distress. Second, the relatively small sample size and low number of CMM events in this subgroup may have reduced the statistical power to detect meaningful associations.

This large-scale prospective cohort study systematically examines the independent effect of the HbA1c/HDL-C ratio and its joint effect with depression on the risk of CMM. The robustness and consistency of the findings were supported by comprehensive subgroup and sensitivity analyses. However, several limitations should be acknowledged. First, although we adjusted for a wide range of known confounders, the possibility of residual confounding cannot be excluded. Important variables such as estimated glomerular filtration rate, antihypertensive medication use, and antidepressant treatment were not available and thus not included in the analysis, which may have influenced the observed associations. Second, the diagnoses of diabetes, heart disease, and stroke were based on self-reported physician diagnoses collected at fixed follow-up intervals, which may have introduced information bias due to recall errors or misclassification. The lack of precise onset dates may also have reduced the accuracy of time-to-event analyses. Third, depression was assessed using the CES-D-10, which is a screening tool rather than a diagnostic instrument. The cutoff value of ≥ 10 has not been formally validated in this study population, which may have introduced misclassification and should be considered when interpreting the results. Finally, while this highlights the public health relevance of multimorbidity, our analysis did not examine individual component diseases of CMM and treated it as a binary outcome, without assessing disease count or severity. Future studies could use disease counts or weighted scores to better capture CMM burden.

Further research in larger, multi-center cohorts that include more diverse populations is needed. Integrating medical records, laboratory tests, imaging data, and detailed information on medication use and lifestyle factors would enhance diagnostic accuracy and provide a more comprehensive understanding of the interplay between metabolic health, mental health, and CMM risk.

## Conclusion

Both the HbA1c/HDL-C ratio and depression are important risk factors for CMM among middle-aged and older adults. Their combined effect significantly increases the risk of CMM, highlighting the need to consider both metabolic indicators and mental health when assessing CMM risk. The findings provide a scientific basis for the early identification and personalized intervention of high-risk populations.

## Data Availability

The datasets presented in this study can be found in online repositories. The names of the repository/repositories and accession number(s) can be found at: https://charls.charlsdata.com/pages/data/111/zh-cn.html.
