# Supplementary material for: Association of HbA1c/HDL-C ratio and depression with cardiometabolic multimorbidity in middle-aged and older adults: a nationwide prospective cohort study
Source: Front Nutr. 2025 Oct 2;12:1642243. doi: 10.3389/fnut.2025.1642243 (PMC12529971; doi:10.3389/fnut.2025.1642243)
Supplement: Supplementary file 1 [file Table_1.DOCX]

**Supplemental Material**

**Content of Supplemental Material**

**Supplemental Tables**

- **Table S1** Responses to the 10-item Center for Epidemiologic Studies Depression Scale.
- **Table S2** Summary of missing value proportions for covariates.
- **Table S3** Assessment of multicollinearity using GVIF and adjusted GVIF.
- **Table S4** Baseline characteristics according to the quartiles of the HbA1c/HDL-C ratio.
- **Table S5** Baseline characteristics according to the depression status.
- **Table S6** Association of HbA1c/HDL-C ratio with the risk of CMM.
- **Table S7** Predictive accuracy of models for CMM across follow-up time points.
- **Table S8** Sensitivity analyses of the joint effects of HbA1c/HDL-C ratio and depression on the risks of developing CMM in participants without diabetes, heart disease, or stroke at baseline (N=4626).
- **Table S9** Sensitivity analyses of the joint effects of HbA1c/HDL-C ratio and depression on the risk of developing CMM after excluding cases that developed CMM in 2013 and 2015 (N=5575).
- **Table S10** Sensitivity analyses of the joint effects of HbA1c/HDL-C ratio and depression on the risks of developing CMM after imputation of missing data (N=9838).

**Supplemental Figures**

- **Fig. S1** Subgroup and interaction analyses of HbA1c/HDL-C ratio and CMM risk.
- **Fig. S2** Mediating effects of depression in the relationship between HbA1c/HDL-C ratio and CMM risk.

**Table S1 Responses to the 10-item Center for Epidemiologic Studies Depression Scale.**

| Item | Response |
| --- | --- |
| 1. I was bothered by things that don't usually bother me. 2. I had trouble keeping my mind on what I was doing. 3. I felt depressed. 4. I felt everything I did was an effort. 5. I felt hopeful about the future. 6. I felt fearful. 7. My sleep was restless. 8. I was happy. 9. I felt lonely. 10. I could not get "going." | 1. Rarely or none of the time (< 1 day) 2. Some or a little of the time (1-2 days) 3. Occasionally or a moderate amount of the time (3-4 days) 4. Most or all of the time (5-7 days) |

**Table S2 Summary of missing value proportions for covariates.**

| Age | Gender | Education |
| --- | --- | --- |
| 0.0% | 0.1% | 0.0% |
| Location | Marital | WC |
| 0.0% | 0.0% | 11.2% |
| BMI | Smoking | LDL-C |
| 11.8% | 0.0% | 0.2% |
| Drinking | Sleep duration | Creatinine |
| 0.0% | 0.5% | 0.3% |
| TC | TG | UA |
| 0.1% | 0.1% | 0.0% |
| CRP | DBP | Kidney disease |
| 0.0% | 11.8% | 0.5% |
| SBP | Hypertension | Antidiabetic |
| 11.8% | 8.4% | 8.2% |
| Antidyslipidemic |  |  |
| 1.9% |  |  |

**Abbreviations:** BMI, body mass index; WC, waist circumference; SBP, systolic blood pressure; DBP, diastolic blood pressure; LDL-C, low-density lipoprotein cholesterol; TC, total cholesterol; TG, triglyceride; UA, uric acid; CRP, C-reactive protein.

**Table S3 Assessment of multicollinearity using GVIF and adjusted GVIF.**

| Characteristics | GVIF | Df | GVIF^(1/(2*Df)) |
| --- | --- | --- | --- |
| Gender | 2.686352 | 1 | 1.639009 |
| Age | 1.514487 | 1 | 1.230645 |
| Location | 1.109371 | 1 | 1.053267 |
| Marital | 1.074809 | 1 | 1.036730 |
| Education | 1.434199 | 3 | 1.061944 |
| BMI | 1.560336 | 1 | 1.249134 |
| WC | 1.475544 | 1 | 1.214720 |
| Smoking | 2.022118 | 2 | 1.192481 |
| Drinking | 1.324251 | 1 | 1.150761 |
| SBP | 3.340290 | 1 | 1.827646 |
| DBP | 2.604961 | 1 | 1.613989 |
| Depression | 1.127718 | 1 | 1.061941 |
| Sleep duration | 1.071417 | 1 | 1.035093 |
| Hypertension | 1.972522 | 1 | 1.404465 |
| Kidney disease | 1.024598 | 1 | 1.012224 |
| Antidiabetic | 1.179595 | 1 | 1.086092 |
| Antidyslipidemic | 1.075124 | 1 | 1.036882 |
| LDL-C | 7.915757 | 1 | 2.813495 |
| TC | 9.226823 | 1 | 3.037569 |
| TG | 3.421268 | 1 | 1.849667 |
| Creatinine | 1.691507 | 1 | 1.300579 |
| UA | 1.592882 | 1 | 1.262094 |
| CRP | 1.018865 | 1 | 1.009388 |
| HbA1c/HDL-C | 2.351764 | 1 | 1.533546 |

**Abbreviations:** GVIF, generalized variance inflation factor; BMI, body mass index; WC, waist circumference; SBP, systolic blood pressure; DBP, diastolic blood pressure; LDL-C, low-density lipoprotein cholesterol; TC, total cholesterol; TG, triglyceride; UA, uric acid; CRP, C-reactive protein; HDL-C, high-density lipoprotein cholesterol; HbA1c, hemoglobin A1c.

**Table S4 Baseline characteristics according to the quartiles of the HbA1c/HDL-C ratio.**

| Characteristics | Q1 (n = 1992) | Q2 (n = 1927) | Q3 (n = 1831) | Q4 (n = 1506) | *P* value |
| --- | --- | --- | --- | --- | --- |
| **Gender, n(%)** |  |  |  |  | <0.001 |
| Female | 1079 (54.17) | 1074 (55.73) | 964 (52.65) | 716 (47.54) |  |
| Male | 913 (45.83) | 853 (44.27) | 867 (47.35) | 790 (52.46) |  |
| **Age (years)** | 59.00 [52.00, 66.00] | 58.00 [51.00, 65.00] | 58.00 [51.00, 64.00] | 58.00 [52.00, 64.00] | 0.002 |
| **Location, n(%)** |  |  |  |  | <0.001 |
| Urban | 562 (28.21) | 635 (32.95) | 642 (35.06) | 629 (41.77) |  |
| Rural | 1430 (71.79) | 1292 (67.05) | 1189 (64.94) | 877 (58.23) |  |
| **Marital, n(%)** |  |  |  |  | <0.001 |
| Single | 360 (18.07) | 297 (15.41) | 268 (14.64) | 193 (12.82) |  |
| Married | 1632 (81.93) | 1630 (84.59) | 1563 (85.36) | 1313 (87.18) |  |
| **Education, n(%)** |  |  |  |  | <0.001 |
| Illiteracy | 603 (30.27) | 564 (29.27) | 481 (26.27) | 338 (22.44) |  |
| Primary school | 830 (41.67) | 796 (41.31) | 763 (41.67) | 656 (43.56) |  |
| Middle school | 383 (19.23) | 380 (19.72) | 388 (21.19) | 335 (22.24) |  |
| High school and above | 176 (8.84) | 187 (9.70) | 199 (10.87) | 177 (11.75) |  |
| **BMI (kg/m^2^)** |  |  |  |  | <0.001 |
| < 24 | 1525 (76.56) | 1248 (64.76) | 969 (52.92) | 624 (41.43) |  |
| ≥ 24 | 467 (23.44) | 679 (35.24) | 862 (47.08) | 882 (58.57) |  |
| **WC (cm)** | 80.00 [74.20, 86.80] | 83.00 [77.00, 89.20] | 86.40 [79.10, 93.00] | 90.00 [83.00, 96.97] | <0.001 |
| **Smoking, n(%)** |  |  |  |  | <0.001 |
| Non-smoker | 1218 (61.14) | 1221 (63.36) | 1105 (60.35) | 846 (56.18) |  |
| Ex-smoker | 142 (7.13) | 153 (7.94) | 157 (8.57) | 176 (11.69) |  |
| Smoker | 632 (31.73) | 553 (28.70) | 569 (31.08) | 484 (32.14) |  |
| **Drinking, n(%)** | 668 (33.53) | 457 (23.72) | 386 (21.08) | 310 (20.58) | <0.001 |
| **SBP (mmHg)** | 125.33 [113.33, 140.67] | 125.33 [112.67, 140.67] | 127.67 [114.67, 142.17] | 127.67 [116.33, 142.33] | <0.001 |
| **DBP (mmHg)** | 73.67 [65.67, 81.67] | 73.67 [66.33, 82.33] | 75.00 [68.00, 84.00] | 76.00 [68.67, 84.33] | <0.001 |
| **Depression, n(%)** | 786 (39.46) | 735 (38.14) | 667 (36.43) | 521 (34.59) | 0.020 |
| **Sleep duration (h)** | 6.00 [5.00, 8.00] | 6.00 [5.00, 8.00] | 7.00 [5.00, 8.00] | 7.00 [5.00, 8.00] | 0.004 |
| **Hypertension, n(%)** | 689 (34.59) | 710 (36.84) | 750 (40.96) | 671 (44.56) | <0.001 |
| **Kidney disease, n(%)** | 141 (7.08) | 120 (6.23) | 108 (5.90) | 99 (6.57) | 0.489 |
| **Antidiabetic, n(%)** | 17 (0.85) | 21 (1.09) | 38 (2.08) | 144 (9.56) | <0.001 |
| **Antidyslipidemic, n(%)** | 52 (2.61) | 68 (3.53) | 84 (4.59) | 143 (9.50) | <0.001 |
| **LDL-C (mmol/L)** | 2.92 [2.43, 3.50] | 3.00 [2.51, 3.63] | 3.04 [2.51, 3.62] | 2.82 [2.24, 3.36] | <0.001 |
| **TC (mmol/L)** | 5.09 [4.53, 5.71] | 4.89 [4.30, 5.55] | 4.83 [4.25, 5.45] | 4.69 [4.15, 5.39] | <0.001 |
| **TG (mmol/L)** | 0.87 [0.67, 1.14] | 1.06 [0.80, 1.43] | 1.33 [0.98, 1.79] | 1.86 [1.29, 2.73] | <0.001 |
| **Creatinine (µmol/L)** | 66.00 [57.00, 76.00] | 66.00 [57.00, 77.00] | 67.00 [58.00, 78.00] | 69.00 [59.25, 80.00] | <0.001 |
| **UA (µmol/L)** | 243.75 [206.00, 294.32] | 248.00 [208.30, 296.85] | 256.40 [215.40, 304.70] | 272.00 [224.93, 324.38] | <0.001 |
| **CRP (mg/L)** | 0.73 [0.43, 1.54] | 0.90 [0.53, 1.86] | 1.08 [0.61, 2.19] | 1.39 [0.72, 2.88] | <0.001 |

**Abbreviations:** BMI, body mass index; WC, waist circumference; SBP, systolic blood pressure; DBP, diastolic blood pressure; LDL-C, low-density lipoprotein cholesterol; TC, total cholesterol; TG, triglyceride; UA, uric acid; CRP, C-reactive protein; HDL-C, high-density lipoprotein cholesterol; HbA1c, hemoglobin A1c.

**Table S5 Baseline characteristics according to the depression status.**

| Characteristics | Non-depression (n =4547) | Depression (n = 2709) | *P* value |
| --- | --- | --- | --- |
| **Gender, n(%)** |  |  | <0.001 |
| Female | 2168 (47.68) | 1665 (61.46) |  |
| Male | 2379 (52.32) | 1044 (38.54) |  |
| **Age (years)** | 57.00 [51.00, 64.00] | 59.00 [53.00, 66.00] | <0.001 |
| **Location, n(%)** |  |  | <0.001 |
| Urban | 1727 (37.98) | 741 (27.35) |  |
| Rural | 2820 (62.02) | 1968 (72.65) |  |
| **Marital, n(%)** |  |  | <0.001 |
| Single | 562 (12.36) | 556 (20.52) |  |
| Married | 3985 (87.64) | 2153 (79.48) |  |
| **Education, n(%)** |  |  | <0.001 |
| Illiteracy | 1070 (23.53) | 916 (33.81) |  |
| Primary school | 1823 (40.09) | 1222 (45.11) |  |
| Middle school | 1062 (23.36) | 424 (15.65) |  |
| High school and above | 592 (13.02) | 147 (5.43) |  |
| **BMI (kg/m^2^)** |  |  | <0.001 |
| < 24 | 2607 (57.33) | 1759 (64.93) |  |
| ≥ 24 | 1940 (42.67) | 950 (35.07) |  |
| **WC (cm)** | 84.80 [78.00, 92.00] | 83.20 [76.00, 90.20] | <0.001 |
| **Smoking, n(%)** |  |  | <0.001 |
| Non-smoker | 2635 (57.95) | 1755 (64.78) |  |
| Ex-smoker | 418 (9.19) | 210 (7.75) |  |
| Smoker | 1494 (32.86) | 744 (27.46) |  |
| **Drinking, n(%)** | 1279 (28.13) | 542 (20.01) | <0.001 |
| **SBP (mmHg)** | 126.67 [114.67, 141.33] | 125.67 [113.33, 142.00] | 0.208 |
| **DBP (mmHg)** | 75.00 [67.33, 83.33] | 74.00 [66.33, 82.67] | 0.005 |
| **Sleep duration (h)** | 7.00 [6.00, 8.00] | 6.00 [4.00, 7.00] | <0.001 |
| **Hypertension, n(%)** | 1723 (37.89) | 1097 (40.49) | 0.030 |
| **Kidney disease, n(%)** | 227 (4.99) | 241 (8.90) | <0.001 |
| **Antidiabetic, n(%)** | 126 (2.77) | 94 (3.47) | 0.108 |
| **Antidyslipidemic, n(%)** | 193 (4.24) | 154 (5.68) | 0.006 |
| **LDL-C (mmol/L)** | 2.95 [2.42, 3.53] | 2.96 [2.42, 3.56] | 0.358 |
| **TC (mmol/L)** | 4.88 [4.31, 5.53] | 4.94 [4.32, 5.57] | 0.100 |
| **TG (mmol/L)** | 1.16 [0.83, 1.69] | 1.16 [0.84, 1.64] | 0.469 |
| **Creatinine (µmol/L)** | 68.00 [59.00, 79.00] | 65.00 [56.00, 75.00] | <0.001 |
| **UA (µmol/L)** | 259.20 [216.80, 312.20] | 245.60 [205.10, 293.20] | <0.001 |
| **CRP (mg/L)** | 1.01 [0.54, 2.02] | 0.97 [0.53, 2.12] | 0.919 |
| **HbA1c/HDL-C** | 3.98 [3.25, 4.91] | 3.85 [3.19, 4.79] | 0.001 |

**Abbreviations:** BMI, body mass index; WC, waist circumference; SBP, systolic blood pressure; DBP, diastolic blood pressure; LDL-C, low-density lipoprotein cholesterol; TC, total cholesterol; TG, triglyceride; UA, uric acid; CRP, C-reactive protein; HDL-C, high-density lipoprotein cholesterol; HbA1c, hemoglobin A1c.

**Table S6 Association of HbA1c/HDL-C ratio with the risk of CMM.**

| Characteristics | Quartiles of HbA1c/HDL-C ratio | | | | *p* for trend* | HbA1c/HDL-C ratio per IQR |
| --- | --- | --- | --- | --- | --- | --- |
|  | Q1 | Q2 | Q3 | Q4 |  |  |
| Cases/N | 59/1992 | 97/1927 | 112/1831 | 151/1506 |  | 419/7266 |
| Model 1, HR (95% CI) | 1.00 (ref) | 1.72 (1.25, 2.38) | 2.10 (1.53, 2.88) | 3.51 (2.60, 4.74) | <0.001 | 1.25 (1.22, 1.29) |
| Model 2, HR (95% CI) | 1.00 (ref) | 1.58 (1.14, 2.19) | 1.72 (1.25, 2.38) | 2.67 (1.94, 3.66) | <0.001 | 1.24 (1.19, 1.29) |
| Model 3, HR (95% CI) | 1.00 (ref) | 1.59 (1.12, 2.25) | 1.67 (1.15, 2.44) | 2.19 (1.44, 3.32) | 0.001 | 1.19 (1.12, 1.27) |

**Abbreviations:** HbA1c, Hemoglobin A1c; HDL-C, high-density lipoprotein cholesterol; CMM, cardiometabolic multimorbidity; IQR, interquartile range; CI, confidence interval; HR, hazard ratio; ref, reference; Q, quartile; BMI, body mass index; WC, waist circumference; SBP, systolic blood pressure; DBP, diastolic blood pressure; LDL-C, low-density lipoprotein cholesterol; TC, total cholesterol; TG, triglyceride; UA, uric acid; CRP, C-reactive protein. Model 1: Crude model; Model 2: Adjusted for age, gender, education, location, marital, BMI, WC, smoking, drinking, SBP, DBP and sleep duration; Model 3: Further adjusted for hypertension, kidney disease, antidiabetic, antidyslipidemic, LDL-C, TC, TG, creatinine, UA and CRP. **p* value for linear trend calculated from category median values.

**Table S7 Predictive accuracy of models for CMM across follow-up time points.**

| Model | Time Point | AUC (95 % CI) | Optimal Cutoff | Sensitivity | Specificity | C-index |
| --- | --- | --- | --- | --- | --- | --- |
| HbA1c/HDL-C | 2 | 0.781 (0.721, 0.837) | 1.124 | 0.700 | 0.748 | 0.736 |
|  | 4 | 0.752 (0.716, 0.788) | 0.733 | 0.804 | 0.586 |  |
|  | 7 | 0.747 (0.724,0.769) | 0.946 | 0.678 | 0.692 |  |
| Depression | 2 | 0.795 (0.731, 0.852 ) | 1.445 | 0.750 | 0.763 | 0.742 |
|  | 4 | 0.760 (0.722, 0.795) | 1.125 | 0.762 | 0.643 |  |
|  | 7 | 0.753 (0.728, 0.777) | 1.258 | 0.675 | 0.710 |  |
| HbA1c/HDL-C+Depression | 2 | 0.796 (0.738, 0.855) | 1.536 | 0.733 | 0.784 | 0.747 |
|  | 4 | 0.765 (0.727, 0.802 ) | 1.256 | 0.697 | 0.701 |  |
|  | 7 | 0.758 (0.735, 0.78) | 1.255 | 0.697 | 0.701 |  |

**Abbreviations:** HbA1c, hemoglobin A1c; HDL-C, high-density lipoprotein cholesterol; CMM, cardiometabolic multimorbidity; AUC, area under the curve; CI, confidence interval; C-index, concordance index. All models were adjusted for age, gender, education, location, marital, BMI, WC, smoking, drinking, SBP, DBP, sleep duration, hypertension, kidney disease, antidiabetic, antidyslipidemic, LDL-C, TC, TG, creatinine, UA, and CRP.

**Table S8 Sensitivity analyses of the joint effects of HbA1c/HDL-C ratio and depression on the risks of developing CMM in participants without diabetes, heart disease, or stroke at baseline (N=4626).**

| Categories | Model 1 | |  | Model 2 | |  | Model 3 | |
| --- | --- | --- | --- | --- | --- | --- | --- | --- |
|  | HR (95% CI) | *P* value |  | HR (95% CI) | *P* value |  | HR (95% CI) | *P* value |
| HbA1c/HDL-C < median without Depression | 1.00 (ref) |  |  | 1.00 (ref) |  |  | 1.00 (ref) |  |
| HbA1c/HDL-C >= median without Depression | 2.40 (1.77, 3.25) | <0.001 |  | 1.88 (1.37, 2.57) | <0.001 |  | 1.57 (1.11, 2.21) | 0.010 |
| HbA1c/HDL-C < median with Depression | 2.14 ( 1.52, 3.00) | <0.001 |  | 2.19 (1.55, 3.09) | <0.001 |  | 2.05 (1.45, 2.90) | <0.001 |
| HbA1c/HDL-C >= median with Depression | 4.18 ( 3.07, 5.68) | <0.001 |  | 3.37 (2.45, 4.63) | <0.001 |  | 2.57 (1.81, 3.65) | <0.001 |

**Abbreviations:** HbA1c, hemoglobin A1c; HDL-C, high-density lipoprotein cholesterol; CMM, cardiometabolic multimorbidity; HR, hazard ratio; ref, reference. Model 1: Crude model; Model 2: Adjusted for age, gender, education, location, marital, BMI, WC, smoking, drinking SBP, DBP, and sleep duration; Model 3: Further adjusted for hypertension, kidney disease, antidiabetic, antidyslipidemic, LDL-C, TC, TG, creatinine, UA, and CRP.

**Table S9 Sensitivity analyses of the joint effects of HbA1c/HDL-C ratio and depression on the risk of developing CMM after excluding cases that developed CMM in 2013 and 2015 (N=5575).**

| Categories | Model 1 | |  | Model 2 | |  | Model 3 | |
| --- | --- | --- | --- | --- | --- | --- | --- | --- |
|  | HR (95% CI) | *P* value |  | HR (95% CI) | *P* value |  | HR (95% CI) | *P* value |
| HbA1c/HDL-C < median without Depression | 1.00 (ref) |  |  | 1.00 (ref) |  |  | 1.00 (ref) |  |
| HbA1c/HDL-C >= median without Depression | 2.67 (1.79, 3.97) | <0.001 |  | 2.14 (1.42, 3.22) | <0.001 |  | 1.72 (1.10, 2.71) | 0.018 |
| HbA1c/HDL-C < median with Depression | 2.24 (1.43, 3.52) | <0.001 |  | 2.29 (1.45, 3.61) | <0.001 |  | 2.15 (1.36, 2.40) | 0.001 |
| HbA1c/HDL-C >= median with Depression | 4.08 (2.70, 6.16) | <0.001 |  | 3.43 (2.24, 5.24) | <0.001 |  | 2.46 (1.54, 3.94) | <0.001 |

**Abbreviations:** HbA1c, hemoglobin A1c; HDL-C, high-density lipoprotein cholesterol; CMM, cardiometabolic multimorbidity; HR, hazard ratio; ref, reference. Model 1: Crude model; Model 2: Adjusted for age, gender, education, location, marital, BMI, WC, smoking, drinking, SBP, DBP, and sleep duration; Model 3: Further adjusted for hypertension, kidney disease, antidiabetic, antidyslipidemic, LDL-C, TC, TG, creatinine, UA, and CRP.

**Table S10 Sensitivity analyses of the joint effects of HbA1c/HDL-C ratio and depression on the risks of developing CMM after imputation of missing data (N=9388).**

| Categories | Model 1 | |  | Model 2 | |  | Model 3 | |
| --- | --- | --- | --- | --- | --- | --- | --- | --- |
|  | HR (95% CI) | *P* value |  | HR (95% CI) | *P* value |  | HR (95% CI) | *P* value |
| HbA1c/HDL-C < median without Depression | 1.00 (ref) |  |  | 1.00 (ref) |  |  | 1.00 (ref) |  |
| HbA1c/HDL-C >= median without Depression | 2.43 (1.90, 3.09) | <0.001 |  | 1.89 (1.48, 2.43) | <0.001 |  | 1.55 (1.18, 2.02) | 0.001 |
| HbA1c/HDL-C < median with Depression | 1.98 (1.50, 2.60) | <0.001 |  | 1.98 (1.49, 2.61) | <0.001 |  | 1.81 (1.37, 3.40) | <0.001 |
| HbA1c/HDL-C >= median with Depression | 3.85 (3.01, 4.93) | <0.001 |  | 3.06 (2.37, 3.95) | <0.001 |  | 2.37 (1.79, 3.13) | <0.001 |

**Abbreviations:** HbA1c, hemoglobin A1c; HDL-C, high-density lipoprotein cholesterol; CMM, cardiometabolic multimorbidity; HR, hazard ratio; ref, reference. Model 1: Crude model; Model 2: Adjusted for age, gender, education, location, marital, BMI, WC, smoking, drinking, SBP, DBP, and sleep duration; Model 3: Further adjusted for hypertension, kidney disease, antidiabetic, antidyslipidemic, LDL-C, TC, TG, creatinine, UA, and CRP.


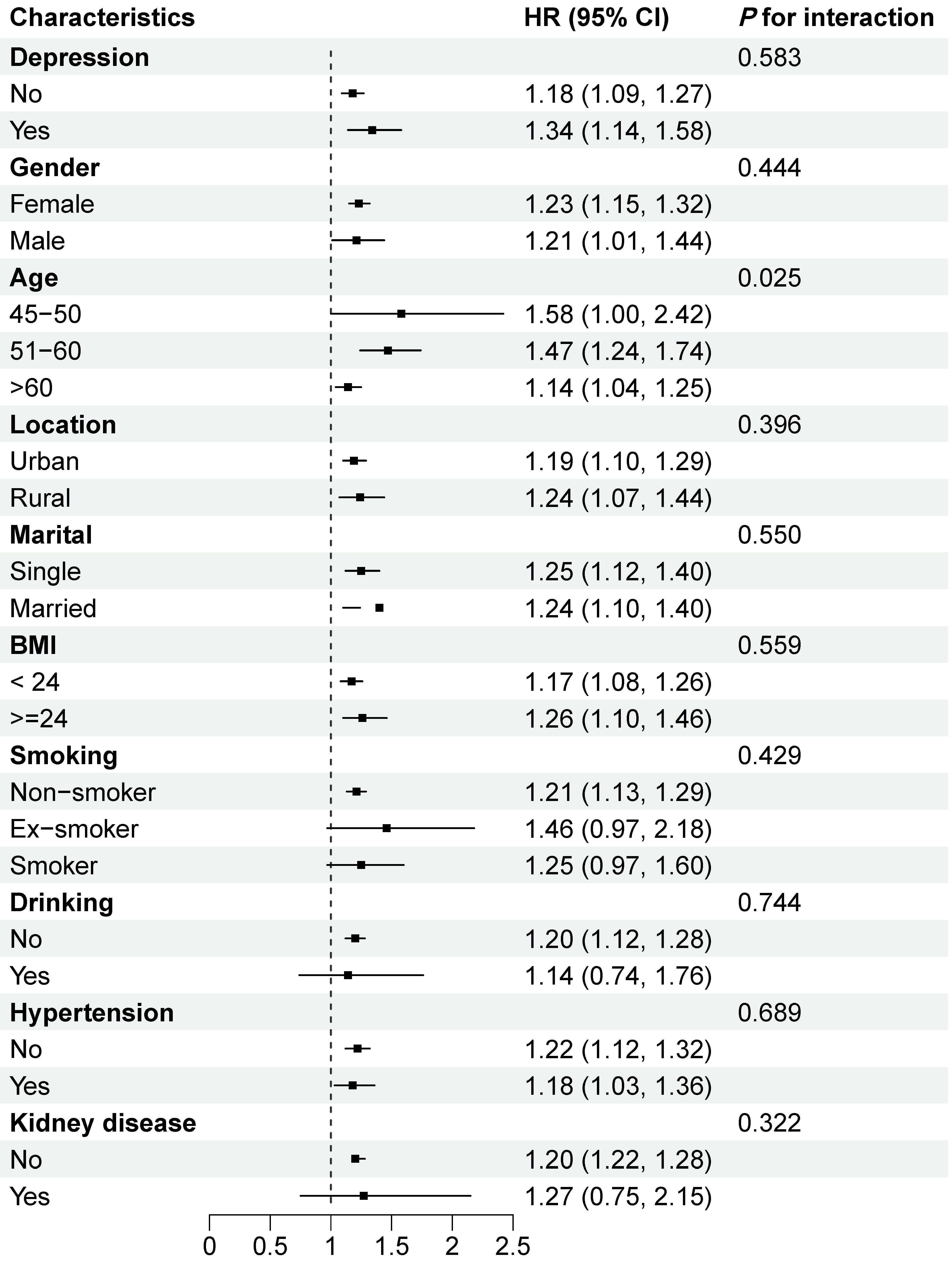


**Fig. S1** Subgroup and interaction analyses of HbA1c/HDL-C ratio and CMM risk. All models were adjusted for age, gender, education, location, marital, BMI, WC, smoking, drinking, SBP, DBP, sleep duration, hypertension, kidney disease, antidiabetic, antidyslipidemic, LDL-C, TC, TG, creatinine, UA, and CRP. Abbreviations: HR, hazard ratio; CI, confidence interval; ref, reference; HbA1c, hemoglobin A1c; HDL-C, high-density lipoprotein cholesterol; CMM, cardiometabolic multimorbidity; BMI, body mass index; LDL-C, low-density lipoprotein cholesterol; TC, total cholesterol; TG, triglyceride; UA, uric acid; CRP, C-reactive protein.


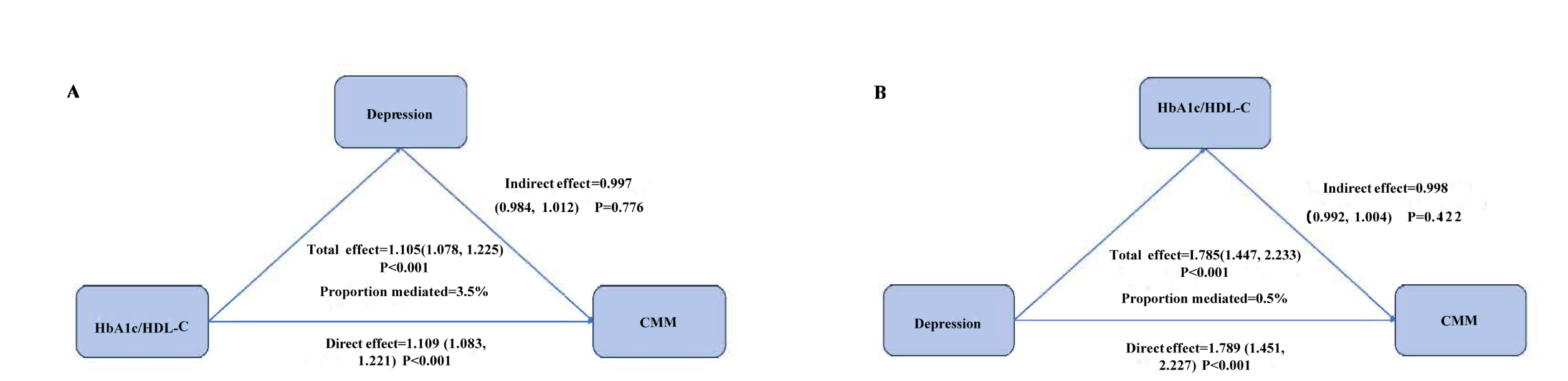


**Fig. S2** Mediating effects of depression in the relationship between HbA1c/HDL-C ratio and CMM risk. Adjusted

for age, gender, education, location, marital, BMI, WC, smoking, drinking, SBP, DBP, sleep duration, hypertension, kidney disease, antidiabetic, antidyslipidemic, LDL-C, TC, TG, creatinine, UA, and CRP. Abbreviations: HbA1c, hemoglobin A1c; HDL-C, high-density lipoprotein cholesterol; CMM, cardiometabolic multimorbidity.
